# Use of Ambulatory Patch Monitoring Devices to Supplement Inpatient Telemetry—A Descriptive Study of a Single-center Experience During the Coronavirus Disease 2019 Pandemic

**DOI:** 10.19102/icrm.2022.130207

**Published:** 2022-02-15

**Authors:** Catherine Vanchiere, Stephen McHugh, Ellen Bedenko, Isaac R. Whitman, Chethan Gangireddy, Anuj K. Basil, Joshua M. Cooper, Daniel Edmundowicz, Meredith Brisco-Bacik, Edmond M. Cronin

**Affiliations:** ^1^Department of Medicine, Department of Medicine, Lewis Katz School of Medicine at Temple University Hospital, Philadelphia, PA, USA; ^2^Section of Cardiology, Department of Medicine, Lewis Katz School of Medicine at Temple University Hospital, Philadelphia, PA, USA; ^3^Global Safety, GlaxoSmithKline, Philadelphia, PA, USA

**Keywords:** Ambulatory monitoring, arrhythmia, COVID-19, QT interval, telemetry

## Abstract

To accommodate the surge in patients with coronavirus disease 2019 during the spring of 2020, outpatient areas in our health system were repurposed as inpatient units. These spaces often lacked the same resources as the standard inpatient unit, including telemetry equipment. We utilized mobile cardiac outpatient telemetry (MCOT) in place of traditional telemetry and suggest that MCOT is an appropriate substitution only for patients at low risk of developing arrhythmia given the prolonged time to notification of the care team regarding events and imprecise measurements of the corrected QT interval when compared to 12-lead electrocardiography.

## Introduction

In 2020, the novel coronavirus disease 2019 (COVID-19) pandemic overwhelmed hospitals across the United States and the world. In Philadelphia County, PA, USA, daily new cases rose to >600 in April 2020 and eventually exceeded that by >10-fold.^1^ One of the many sequelae of COVID-19 is the development of cardiac complications, including arrhythmia, with incidence rates reported to be as high as 16%–20%.^2,3^

Our health system converted outpatient spaces into COVID-19 care units to accommodate up to 200 COVID-19–positive patients at the initial peak of the pandemic and to sequester suspected or confirmed cases in a certain area. Standard telemetry was lacking in these areas, however, so we utilized mobile cardiac outpatient telemetry (MCOT) (BioTelemetry, Malvern, PA, USA) in its place.^4^

## Methods

Patients were stratified by the level of care (intensive care vs. medicine ward service), and patients with a lower severity of illness were selected for the use of MCOT in place of telemetry where telemetry availability was limited. We used MCOT only in lower-risk, non–critically ill patients as determined by a “may transport off telemetry” designation as per the treating physician, which is the standard for non–intensive care unit patients within our system. At the time of MCOT application, a baseline transmission was sent, including data on heart rhythm, heart rate, and baseline corrected QT (QTc) interval as well as a 6-second rhythm strip. Standardized notification criteria were used. Emergency alerts were generated for cases of a narrow complex tachycardia of >220 bpm, bradycardia of <20 bpm, ventricular tachycardia, or ventricular fibrillation. Urgent alerts were generated when any of the following were observed: a QTc of >500 ms, pause/asystole of >3 seconds, syncope, a narrow complex tachycardia lasting >30 seconds, a case of bradycardia of less than 35 bpm, a case of new-onset atrial fibrillation/flutter (AF/AFL) lasting >30 seconds, nonsustained ventricular tachycardia, any second-degree Mobitz type II or third-degree heart block, or failure of a defibrillator/pacemaker to capture/sense. Alerts were communicated from an MCOT representative by phone to nursing personnel and emailed to an electrophysiologist for review. The time of notification delivery was documented in the end-of-service summary. The QTc was manually measured by the vendor’s monitoring technicians and reported in the baseline transmission, daily rhythm strip, and together with any captured sinus rhythm events reported in the end of service summary.

For the purposes of this study, only patients who were confirmed to be COVID-19–positive were included for evaluation. We utilized MCOT only where necessary to make up for a lack of standard telemetry. We retrospectively reviewed all MCOT alerts and reports for each of these patients. We retrospectively compared the MCOT QTc measurement with 12-lead electrocardiograms (ECGs) recorded within 48 hours of the baseline transmission, overread by a cardiac electrophysiologist using digital calipers (MUSE; GE Healthcare, Chicago, IL, USA). MCOT was discontinued upon transfer to a non–COVID-19 floor. The Temple University Institutional Review Board approved this study.

## Results

Twenty-three patients were monitored with MCOT; 3 were found to be COVID-19–negative and hence were transferred to standard telemetry units. **Table 1** summarizes the indications and clinical data of the COVID-19–positive patients (n = 20). Their mean age was 58.1 ± 14.2 years, 45% were men, and 70% were Black or African American. On presentation, 90% of the patients were in sinus rhythm and 10% were in AF. While many of our patients received azithromycin (85%), 2 received hydroxychloroquine and azithromycin (10%), and no patients received hydroxychloroquine alone.

Seven of the 20 COVID-19–positive patients generated MCOT alerts, totaling 12 events captured over 992 cumulative hours of monitoring (mean, 124 hours; range, 2–431 hours). Six of the events were for AF/AFL, 2 were for nonsustained ventricular tachycardia, 2 were for severe bradycardia, 1 was for a QTc interval of >500 ms, and 1 was for second-degree atrioventricular block, respectively. On review of the AF/AFL alerts by a cardiac electrophysiologist, 4 of the 6 events were found in patients with known AF and 2 were false, with no AF seen upon review of the transmissions. Of the 12 events, only 1—severe bradycardia of >35 bpm, which was a terminal rhythm in a patient who had been upgraded to intensive care and placed on telemetry—was immediately actionable.

Verbal notification to the care team was unsuccessful in 1 event, and, for another event, there was no recorded time of notification. The average time to notification (n = 10) was 4 hours and 7 minutes (range, 16 minutes–22 hours and 6 minutes). For 6 events, the notification was delivered within one hour.

Of the 23 patients initially monitored with MCOT, 16 had a 12-lead ECG within 48 hours of the baseline transmission. The mean difference between the QTc interval measured on ECG and MCOT was an absolute value of 31 ms (± 20.9 ms; minimum, 3 ms; maximum, 76 ms). Eight patients had a longer QTc interval on MCOT and 8 patients had a longer QTc interval on ECG, respectively. Two patients had a difference of <10 ms between MCOT and ECG, but 50% of patients had a ≥30-ms difference.

## Discussion

Patch ECG monitoring was initially created as an alternative to Holter monitoring with an increased ease-of-use profile for longer-term ambulatory monitoring in outpatients. Compared to Holter monitoring, patch ECG monitoring allows for longer wear by patients and has demonstrated an increased rate of detection for certain arrhythmias.^5–7^ The primary indications for patch ECG monitoring include detection of paroxysmal arrhythmias and the assessment of arrhythmia burden. The United States Food and Drug Administration extended the clearance to include analysis and reporting of QTc intervals during March 2020 for both MCOT and KardiaMobile (AliveCor, Mountain View, CA, USA), a 6-lead personal ECG technology.^8,9^

Despite this clearance, however, the data regarding the accuracy of QTc measurement among ambulatory monitors remain uncertain. A prior single-center study that examined the use of MCOT for QTc interval monitoring in COVID-19 patients on hydroxychloroquine and azithromycin found that the use of MCOT increased the ease of monitoring QTc without serial ECGs or standard telemetry.^10^ The authors did not, however, compare QTc measurements from MCOT to those from 12-lead ECGs, nor did they quantify the impact of a reduction in patient interactions, and they concluded that MCOT should be used in patients with greater comorbidities with caution. The measurement of the QTc interval with a single-lead handheld ECG (Kardia; AliveCor) was tested and found to have increased reliability with use at multiple lead locations.^11^ In particular, the QTc interval measured at any single lead position (lead I, lead II, precordial) was found to be shorter than that measured on a 12-lead ECG, while the use of the maximal QTc interval across multiple lead positions was more accurate. Another study looking at QT-interval measurement with the Apple Watch (Apple Inc., Cupertino, CA, USA) confirmed a difference of ≤20 ms between QT-interval measurements made by a 12-lead ECG versus the Apple Watch at any of the 3 lead positions.^12^ Our study shows a high variation in QTc-interval measurements between a 12-lead ECG and MCOT, with a mean difference of 31 ms. We did not utilize multiple lead positions given the goal of reducing patient interactions within COVID-19 units. Likewise, 12-lead ECGs were not obtained at the time of baseline MCOT transmission and were included for study if collected within 48 hours of MCOT placement. This timing difference may account for some of the variation we see in our cohort.

Evaluation of offsite cardiac monitoring of non–critically ill inpatients, such as that utilized with MCOT in this study, has been performed in large cohorts and validated as a safe alternative to traditional onsite monitoring.^13^ In one study by Cantillon et al., 79% (772/979) of the notifications for rate/rhythm changes that were followed by activation of the emergency response team within 1 hour were accurate.^13^ Within our small cohort, only 50% of events were followed by an accurate notification within 1 hour, with the average time to notification exceeding 4 times as long. The reasons behind notification delays are not clear, but the lack of a protocol or familiarity with remote monitoring for inpatients may have contributed to the delays.

The use of MCOT in COVID-19 patients may offer the benefit of the reduced need for nursing interaction to fix displaced leads or electrodes, thus decreasing patient interactions and the use of personal protective equipment (PPE). Particularly early on in the pandemic, when many health centers struggled to provide adequate staff and adequate PPE for safe patient care, these purported benefits were particularly valuable. The weaknesses of MCOT may include poor reliability for the measurement of the QTc interval among inpatients, as there is a poor consensus within the current literature. Furthermore, utilizing multiple lead positions as described in other studies would eliminate the benefit of minimizing staff contact. While this study does not directly compare the time to notification between MCOT and traditional telemetry, it does demonstrate that the time to notification in this small cohort was quite long and variable compared to that in prior studies performed on a larger scale, thus making the use of MCOT for patients with a high risk of harmful or fatal arrhythmia potentially unsafe.

Our findings highlight the importance of triaging the use of patch ECG monitoring thoroughly to select patients in whom its use is effective and safe. We propose that inpatients at risk of prolonged QTc intervals should undergo both 12-lead ECG and traditional telemetry in order to ascertain accurate and reliable values. Patients with a low risk of QTc-interval prolongation or who are being monitored for paroxysmal arrhythmias are likely to do well with MCOT use as an alternative to telemetry, but major risk factors, such as severe electrolyte derangement or the use of continuous inotropic agents, should prompt immediate transition to standard telemetry.

The limitations of this study include its small sample size and single-center, nonrandomized design. While we did not directly compare the frequency of actionable alarms or the notification time for telemetry alarms to those of MCOT, the response times seen in our cohort were slower than the previously published times. Additionally, QTc-interval measurements on ECG and MCOT were not made simultaneously, which would have required additional staff exposure to patients and use of PPE.

## Conclusions

Our findings suggest that MCOT may be a reasonable alternative to telemetry in lower-risk inpatients, but the long time to notification and imprecise QTc-interval measurements support the continued use of standard telemetry for inpatients at risk of life-threatening arrhythmias.

## Figures and Tables

**Table 1:** Indications and Clinical Data for COVID-19–positive Patients (n = 20)

	Category	n (%)
Indication(s) for MCOT^a^		
	Troponin elevation	3 (15)
	QT prolonging Rx	3 (15)
	Hx of CAD/HF	5 (25)
	Hx of arrhythmia	5 (25)
	New arrhythmia	4 (20)
	Other	6 (30)
Medical history		
	Hypertension	15 (75)
	Heart failure	8 (40)
	AF/AFL	5 (25)
	Coronary artery disease	7 (35)
	Diabetes mellitus	9 (45)
	Hyperlipidemia	12 (60)
	Stroke	2 (10)
	Obstructive sleep apnea	5 (25)
COVID-19 treatments^b^		
	Azithromycin	17 (85)
	Remdesivir	2 (10)
	Immune-modulating agents	10 (50)
	Intravenous immunoglobulin	3 (15)
	Corticosteroids	19 (95)
	Hydroxychloroquine	2 (10)
	Convalescent plasma	2 (10)
Cardiac medications^b^		
	Aspirin	7 (35)
	Statin	8 (40)
	β-blocker	8 (40)
	ACEI/ARB	5 (25)
	CCB	6 (30)
	Diuretics	7 (35)
	Anticoagulation	3 (15)
Admission laboratory values		
	BNP > 100 pg/mL	17/17 (100)
	cTnI > 0.045 ng/mL	6/19 (31.6)
	D-dimer > 230 ng/mL	4/15 (26.7)

*Abbreviations*: ACEI, angiotensin-converting enzyme inhibitors; AF, atrial fibrillation; AFL, atrial flutter; ARB, angiotensin receptor blocker; BNP, brain natriuretic peptide; CAD, coronary artery disease; CCB, calcium channel blocker; COVID-19, coronavirus disease 2019; cTnI, cardiac troponin I; HF, heart failure.

^a^More than one indication was selected for some patients.

^b^Reflects medications administered during hospitalization.
